# Impact of Fluoride Exposure on Rat Placenta: Foetal/Placental Morphometric Alterations and Decreased Placental Vascular Density

**DOI:** 10.1007/s12011-023-03916-5

**Published:** 2023-10-26

**Authors:** Jonathan Guerrero-Arroyo, Mónica I. Jiménez-Córdova, Octavio G. Aztatzi-Aguilar, Luz M. Del Razo

**Affiliations:** Departamento de Toxicología, Centro de Investigación y de Estudios Avanzados del Instituto Politécnico Nacional, 07360 México City, México

**Keywords:** Fluoride, Morphometrics, Pregnancy, Placental abnormalities, Vascular density, Placental weight

## Abstract

Inorganic fluoride is a geogenic and anthropogenic contaminant widely distributed in the environment and commonly identified in contaminated groundwater. There is limited information on the effect of fluoride exposure on pregnancy. The aim of this study was to evaluate possible placental alterations of fluoride exposure in a rat model simulating preconception and pregnancy exposure conditions in endemic areas. Fluoride exposure was administered orally to foetuses of dams exposed to 2.5 and 5 mg fluoride/kg/d. Foetal weight, height, foetal/placental weight ratio, placental zone thickness, levels of malondialdehyde (MDA) and vascular endothelial growth factor-A (VEGF-A) and vascular density in placental tissue were evaluated. The results showed a nonlinear relationship between these outcomes and the dose of fluoride exposure. In addition, a significant increase in the fluoride concentration in placental tissue was observed. The group that was exposed to 2.5 mg fluoride/kg/d had a greater increase in both MDA levels and VEGF-A levels than the higher dose group. A significant increase in the thickness of the placental zones and a decrease in the vascular density of the labyrinth zone area were also observed in the fluoride-exposed groups. In conclusion, the data obtained demonstrate that fluoride exposure results in morpho-structural alterations in the placenta and that non-monotonic changes in MDA, VEGF-A levels and placental foetal weight ratio were at environmentally relevant concentrations.

## Introduction

Inorganic fluoride is ubiquitous in various geological environments and can reach hazardous concentrations in groundwater and soils due to geochemical processes [1]. Globally, there are many endemic areas in low- and middle-income countries in Africa, Asia and Latin America with high exposure to fluoride from groundwater, with approximately 180 million people potentially affected worldwide [2]. In these countries with fluoride contamination problems in water, the following have been reported: damage to renal physiology [3, 4], decreased cognitive function in children of women exposed to fluoride during pregnancy [5], an increased risk of premature birth and low birth weight [6], and an increased risk of abortion in areas with high concentrations of fluoride [7]. Likewise, alterations in oxidative stress markers [8, 9] and decreased fertility in rats [10, 11] have been reported; however, information on placental alterations caused by fluoride exposure is very limited. However, there is available evidence indicating that fluoride in the mother’s blood crosses the placenta and is absorbed and excreted by the foetus [12]. The placenta is the temporary organ that fulfils the functions of hormone secretion, metabolic transfer, gas exchange and foetal protection and, therefore, is one of the organs most exposed to contaminant damage [13].

Nutrient transfer depends on the physical properties of the placenta [14]. Some studies suggest that fluoride exposure may indirectly affect placental function through effects on the reproductive and endocrine systems in female rats [11], while oxidative stress [15] and placental abnormalities, such as inflammation, hypercoiling and chronic villitis, may also contribute to placental insufficiency, having significant effects on pregnancy and foetal development [16].

Knowledge of how fluoride exposure affects placental development is limited; therefore, we hypothesized that fluoride affects placental structure and morphology, which impacts neonatal weight. A dysfunctional placenta can have a negative impact on neonatal weight, and it is important to monitor placental health during pregnancy to prevent adverse outcomes [17]. The objective of this work was to evaluate potential placental alterations caused by fluoride exposure in a rat model that simulates preconception and pregnancy exposure conditions in endemic areas.

## Materials and Methods

### Experimental Design

Fifteen female and eight male Wistar rats (250 ± 31.66 g) were obtained and maintained in the Laboratory Animal Experimentation and Production Unit (UPEAL-CINVESTAV) in accordance with NOM-062-ZOO-1999. The procedures were approved by the Internal Committee for the Care and Use of Laboratory Animals (CICUAL) of CINVESTAV; protocol number 0292-19. The animals were maintained in rooms at a constant temperature (21 ± 2 °C) humidity of 40–60%, with filtered air at 95% efficiency and noise level lower than 85 dB. Rats were provided with free access to rodent food (PicoLab® Mouse Diet 20, #5058, LabDiet®; Haward, CA) and purified water containing 10 μg fluoride per litre. Male rats without any treatment were used for mating.

### Grouping and Fluoride Exposure

**Fig. 1:**
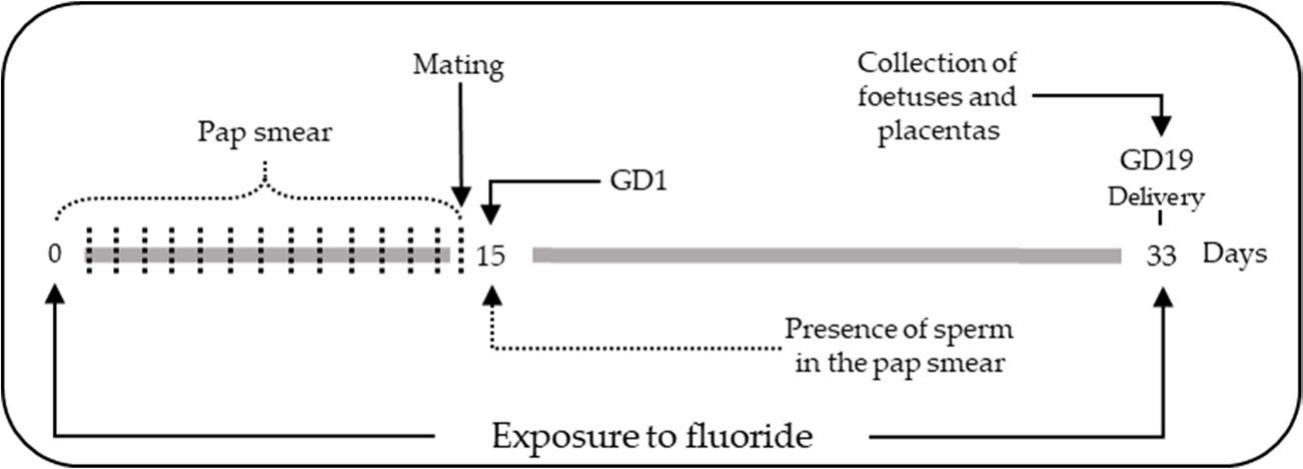
Experimental design of rat exposure to fluoride. Wistar rats were exposed to fluoride by gastric gavage for 33 days. The administered dose was purified water, 2.5 and 5 mg fluoride/kg/d by gavage. The Pap smear was evaluated daily before mating. The presence of sperm in the Pap smear represented gestational day 1 (GD1); GD19, the delivery was performed.

After 8 days of acclimatization, female rats were randomly assigned to three experimental groups (*n* = 5) as follows: control (received 2 ml/kg body mass of purified water by gavage); two exposed groups to sodium fluoride (Sigma–Aldrich, 99% pure) at doses equivalent to 2.5 and 5 mg fluoride/kg/d by gavage for 14 days. At day 14, the females and no exposed males were placed in the same cage at a ratio of 1:2; Pap smear was evaluated and the female rats with sperm observed in vaginal smears under a microscope were regarded as pregnant at day 1 gestation. The control and fluoride exposure group rats continued their treatment for 19 days after mating. Body weight, water intake and food consumption were monitored every 2 days. On gestation day 19 (GD19), under anaesthesia with ketamine/xylazine (80–5 mg/kg) intramuscularly, dystocic delivery was performed, and the gravid oviducts were extracted, from which the foetus and placenta were obtained (Fig. 1). The number, weight and height were recorded. The pregnant rats were euthanized postpartum. Placentas were stored at −70 °C until used for analysis.

### Quantification of Fluoride in the Placenta

The placenta was homogenized by an Omni TH tissue homogenizer (PerkinElmer©) with Hard Tissue Omni Tip Plastic Homogenizing Probes (7×110 mm) (PerkinElmer©) at 35,000 rpm (500 mg in 1 mL of deionized water). The fluoride level in the placenta was evaluated in 25 placentas of each group after overnight hexamethyldisiloxane (HMDS)-facilitated diffusion [18], using a selective ion electrode (OrionTM Fluoride 9409BNWP, Thermo Fisher Scientific Inc.) coupled to a potentiometer. Standard reference material (PC-U-F2102) from the National Institute Public Health of Quebec (0.45 μg fluoride/mL) was prepared in duplicate and diffused in a manner like the samples. The samples and standard graph were prepared in equal proportions with TISAB II (total ionic strength adjustment buffer) solution. The mV reading was converted to μg fluoride using a standard graph. The limit of detection of fluoride in the placenta was 0.010 μg/g, the coefficient of variation was 13.25% and the accuracy was 101.47%.

### Assessment of Malondialdehyde Levels in Placenta

A portion of placenta (25 mg) was homogenised by an Omni TH tissue homogenizer (PerkinElmer©) with Hard Tissue Omni Tip Plastic Homogenizing Probes (7×110 mm) (PerkinElmer©) at 35,000 rpm, on Nonidet-P40 lysis buffer (150 mM NaCl, 1% Triton X-100, 50mM Tris-HCl, pH 8.0) and COMPLETE protease inhibitors; the samples were sonicated for 10 s, centrifuged at 14,000 rpm for 10 min, and the supernatant was used for determination. Both the supernatant of the homogenate and the malondialdehyde (MDA) standard graph were processed in triplicate as indicated in the Thiobarbituric Acid Reactive Substances (TBARS) Assay kit (Cayman, chemical Item No.10009055). As a positive control, 50 μL of 3% hydrogen peroxide (H_2_O_2_) was added to 25 mg of placenta. The absorbance was read at 535 nm. The MDA concentration was expressed as μM per gram protein and calculated from a standard graph.

### Stereological Analysis

Three random rat placentas from each group were evaluated and fixed in formaldehyde in 10% PBS buffer. Thereafter, the placentas were cut to obtain two half parts along the midline through the umbilical cord, and sections at 5 μm were obtained in semiseries, using one in every 60 sections, to avoid repetitive analysis of the same histological area. Cuts were fixed and stained with haematoxylin & eosin (H&E). Stained sections were captured with 4×, 10× and 40× magnifications using the High Resolution Light Optical Microscope Keyence BZ-X800 (Keyence Corporation of America, IL, USA) to produce full panoramic views.

To determine the central placental thickness, a central line was drawn perpendicular to the point of insertion of the umbilical cord, which crosses the entire placental sample, and then two lines were drawn parallel to it, one on each side, with approximately 800 μm. In the same way, the same three lines are drawn for evaluation of the labyrinth zone (LZ). The thickness of the LZ, the basal decidua (BD), the basal zone (BZ), the area of the LZ and the area of glucogenic cells (GC) were also measured using ImageJ v1.54 software, (National Institutes of Health, USA). Finally, vascular density was expressed as the ratio between the total number of vessels counted and the area of the LZ (vessels/mm^2^).

### Evaluation of Vascular Endothelial Growth Factor in the Placenta

Placental homogenate was used to determine the vascular endothelial growth factor (VEGF-A) level. VEGF-A concentration was measured using the rat VEGF-A ELISA kit (ThermoFisher, RAT83785) according to the manufacturer’s instructions. Briefly, samples were warmed to room temperature (RT) and gently agitated, 100 μL of sample/standard were added to the wells with diluents and incubated at RT for 1 h. The wells were then washed, and the plates were incubated at RT for 1 h. The plates were then washed 5 times with a wash buffer and incubated with VEGF conjugate for 1 h at RT. After the washes, the plates were incubated with the substrate solution for 30 min at RT; the reaction was terminated with a stop solution and the absorbance was measured at 450 nm. VEGF-A concentration was expressed as pg/mg of protein and calculated from a standard four-parameter algorithm plot.

### Statistical Analysis

Data are expressed as the median and interquartile range (IQR) or mean values ± standard deviation (SD). A one-way ANOVA or nonparametric test (Kruskal–Wallis test) was performed to determine the significance between groups. Significant value *p* ≤ 0.05; Tukey`s post hoc test was used to determine which groups were significantly different. All statistical analyses were performed using GraphPad Prism software version 8.0.2 (Boston, MA).

## Results

### Dam Gestational Parameters and Fluoride Exposure

After exposure, no significant differences in dam body weight were observed between the control and fluoride exposure groups (Table 1). The area under the curve (AUC) was calculated to assess the body weight gain of each rat during the time of exposure, and no differences were observed. In summary, our results show that fluoride exposure did not significantly affect the dam gestational parameters evaluated.

**Table 1:** Descriptive statistics of dams and foetuses exposed to fluoride

	Control	2.5 mg fluoride/kg/d	5 mg fluoride/kg/d
Dams parameters [Median (IQR)]	*n* = 5	*n* = 5	*n* = 5
Weight (g)	300.1 (280.7–328.3)	311.8 (291.0–335.9)	297.9 (279.1–326.4)
AUC gain weight	128 (126.0–149.5)	128 (91.0–146.5)	118 (97.5–158.0)
Litter size	15 (13–15)	11 (6.5–15.5)	11 (6.5–15.5)
Litter parameters	*n* = 71	*n* = 55	*n* = 55
Weight (g)	1.57 (1.50–1.64)	1.46 (1.38–1.53)^a^	2.24 (1.61–2.56)^b^
Size (cm)	2.55 (2.48–2.62)	2.43 (2.39–2.49)^a^	2.88 (2.60–3.19)^a^
Placenta	*n* = 71	*n* = 55	*n* = 55
Weight (g)	0.47 (0.41–0.50)	0.51 (0.47–0.56)^a^	0.50 (0.44–0.55)^c^
Size (mm)	13.50 (12.92–13.87)	13.68 (13.19–14.18)	13.50 (12.86–14.18)
Foetal/placenta weight ratio	3.4 (3.1–3.7)	2.9 (2.6–3.1)^a^	4.0 (3.2–5.0)^d^
Fluoride concentration (mean ± SD)	*n* = 25	*n* = 25	*n* = 25
Placenta (μg F/g of tissue)	<LOD	0.395 ± 0.052^&^	0.943 ± 0.155^&^

The results are expressed as the median and IQR, ^a^*p* < 0.001, ^b^*p* < 0.01, ^c^*p* < 0.05 significantly different from the control group (Kruskal–Wallis test). Fluoride concentration exposure is expressed as the mean ± SD, ^&^*p* < 0.001 significantly different from the control group (Student’s *t* test). *AUC* área under curve, *LOD* level of detection 0.01 µg fluoride/g, *IQR* ínter quartile range, *SD* standard deviation.

To examine the levels of fluoride that reached placental tissue, quantification of fluoride was conducted. The results showed a correspondence between fluoride exposure dose and fluoride concentration in placental tissue, finding a statistically significant increase in the mean fluoride quantity in the 5 mg fluoride/kg/d exposure group compared with the 2.5 mg fluoride/kg/d exposure group (Table 1). No detectable concentrations of fluoride were observed in the control group (<0.01 μg fluoride/g).

### Foetus Parameters and Exposure Effects

**Fig. 2:**
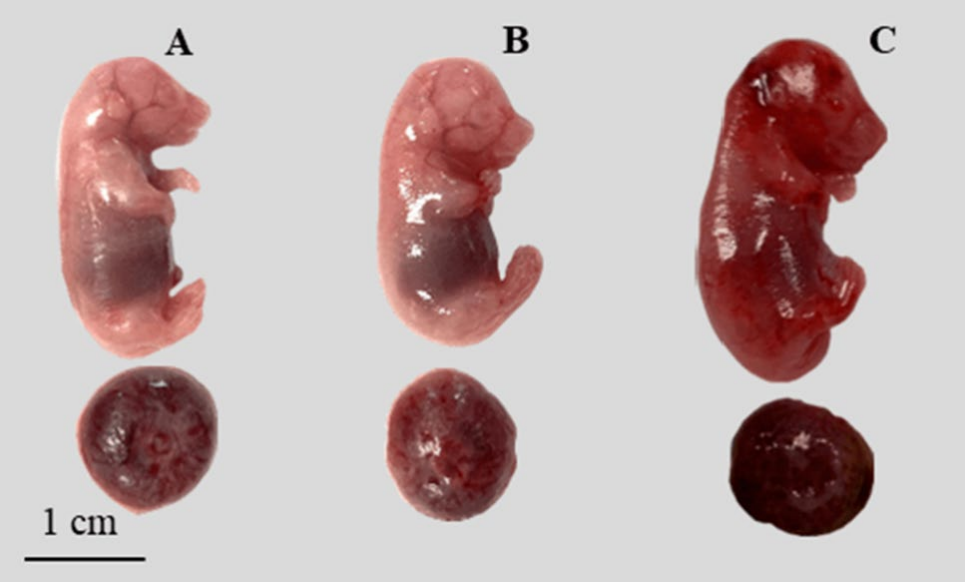
Representative foetuses and placentas from the litter on GD 19. **A** Foetus and placenta of the control group; **B** foetus and placenta treated with 2.5 mg fluoride/kg/d; **C** foetus and placenta treated with 5 mg fluoride/kg/d, foetus with apparent erythema. Bar = 1 cm

Representative foetuses of the control and exposure groups are shown in Fig. 2. Apparent local erythema was observed in foetuses from dams exposed to 5 mg fluoride/kg/d (Fig. 2C) compared with the control and 2.5 mg fluoride/kg/d groups (Fig. 2A, B), in which foetuses showed a uniform pale pink colouration. A statistically significant decrease in the median weight (*p* < 0.0001) and height (*p* < 0.0001) of foetuses was observed in the 2.5 mg fluoride/kg/d exposure group compared with the control (Table 1), while foetuses in the 5 mg fluoride/kg/d exposure group showed a significant increase in weight (*p* < 0.001) and height (*p* < 0.0001).

### Foetal-Placental Weight Relationship

**Fig. 3:**
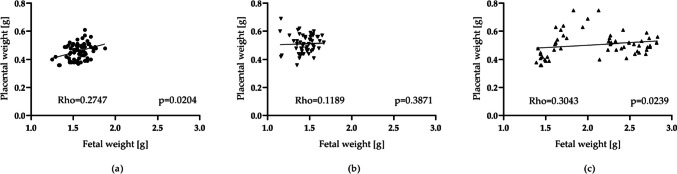
Correlation between foetal and placental weight. **a** Control group; **b** 2.5 mg fluoride/kg/d group; **c** 5 mg fluoride/kg/d group

To investigate placental efficiency, we analysed the relationship between foetal and placental weight in each group. As shown in Fig. 3, a positive and statistically significant correlation between placental and foetal weight was observed in the control group (Fig. 3A) and 5 mg fluoride/kg/d exposure group (Fig. 3C), while no significant correlation was observed in the 2.5 mg fluoride/kg/d exposure group (Fig. 3B). In addition, the foetal/placental weight ratio was significantly higher (*p* < 0.05) in the 5 mg fluoride/kg/d exposure group than in the control group and was lower in the 2.5 mg fluoride/kg/d exposure group (*p* < 0.0001) (Table 1).

### MDA Levels in Placenta

**Fig. 4:**
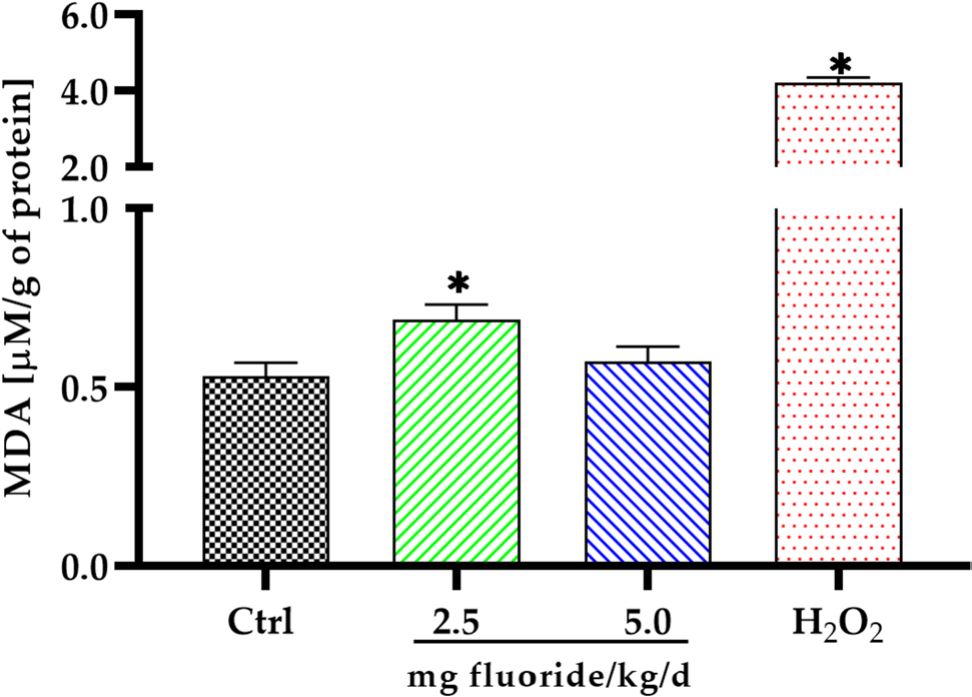
Malondialdehyde (MDA) concentration in placenta. Bars represent mean ± SD of MDA concentration (μM) per gram of protein; hydrogen peroxide (3% H_2_O_2_) was used as positive control for MDA test; *n* = 15 placentas per group, experiments were performed in triplicate; *p < 0.01 significantly different by control group (one-way ANOVA test with post hoc Tukey test)

To explore whether oxidative damage was produced in the placenta by fluoride exposure, levels of MDA, a recognized measure of lipid peroxidation, were analysed. Non-monotonic results were obtained in the concentration of MDA in the placental (Fig. 4). In the 2.5 mg fluoride/kg/d group, a statistically significant increase (*p* < 0.0001) in the concentration of MDA was observed compared with the control group. In contrast, exposure to 5 mg fluoride/kg/d did not significantly affect (*p* = 0.0689) the MDA concentration in the placenta.

### Placental Morphological Effects

To further investigate whether fluoride exposure is associated with placental alterations, morphological characteristics were evaluated, and the results are shown in Table 2. H&E-stained sections showed an increase (*p* < 0.05) in the width of the placenta from rats exposed to 5 mg fluoride/kg/d compared to the control group (3.5 vs. 3.2 mm), without significant changes in placental length. In the histological sections, the thickness of the BD and the LZ increased in the fluoride exposed groups, being significant [(*p* < 0.0001) and *p* < 0.001, respectively] in the 5 mg fluoride/kg/d group, while the BZ was significantly increased (*p* < 0.01) in the 2.5 mg fluoride/kg/d group. Furthermore, in the fluoride-treated groups, no differences were found in both the area of the glycogenic cell clusters and the area of the LZ (Table 2).

**Table 2:** Morphological characteristics of placentas in rats exposed to fluoride

	Control	2.5 mg fluoride/kg/d	5 mg fluoride/kg/d
Placental Dimensions^a^ (mm)			
Length (*n* = 8)	9.9 (9.3–10.9)	9.7 (9.36–10.74)	10.3 (9.39–10.47)
Wide (*n* = 8)	3.2 (3.1–3.3)	3.2 (3.08–3.34)	3.5 (3.27–3.75)*
Width of the placental zones^a^ (mm)			
Basal decidua (*n* = 45)	0.099 (0.08–0.11)	0.117 (0.08–0.16)	0.162 (0.11–0.22)*
Basal zone (*n* = 15)	0.504 (0.40–0.57)	0.604 (0.48–0.75)*	0.462 (0.36–0.56)
Labyrinth zone (*n* = 15)	2.352 (2.29–2.47)	2.375 (2.26–2.34)	2.698 (2.65–2.80)*
Others			
CG cluster area^a^ (*n* = 42) (mm^2^)	11.0 (7.00–19.00)	9.0 (5.00–19.00)	12.5 (8.00–20.75)
LZ area^b^ (*n* = 4) (mm^2^)	18958 ± 1680	18339 ± 1182	19551 ± 1678

*CG* glycogenic cells; *LZ* labyrinth zone

*p < 0.01 significantly different from the control group (Kruskal–Wallis test)

^a^Results expressed as median and inter quartile range (IQR)

^b^The results are expressed as the mean ± SD (one-way ANOVA test)

**Fig. 5:**
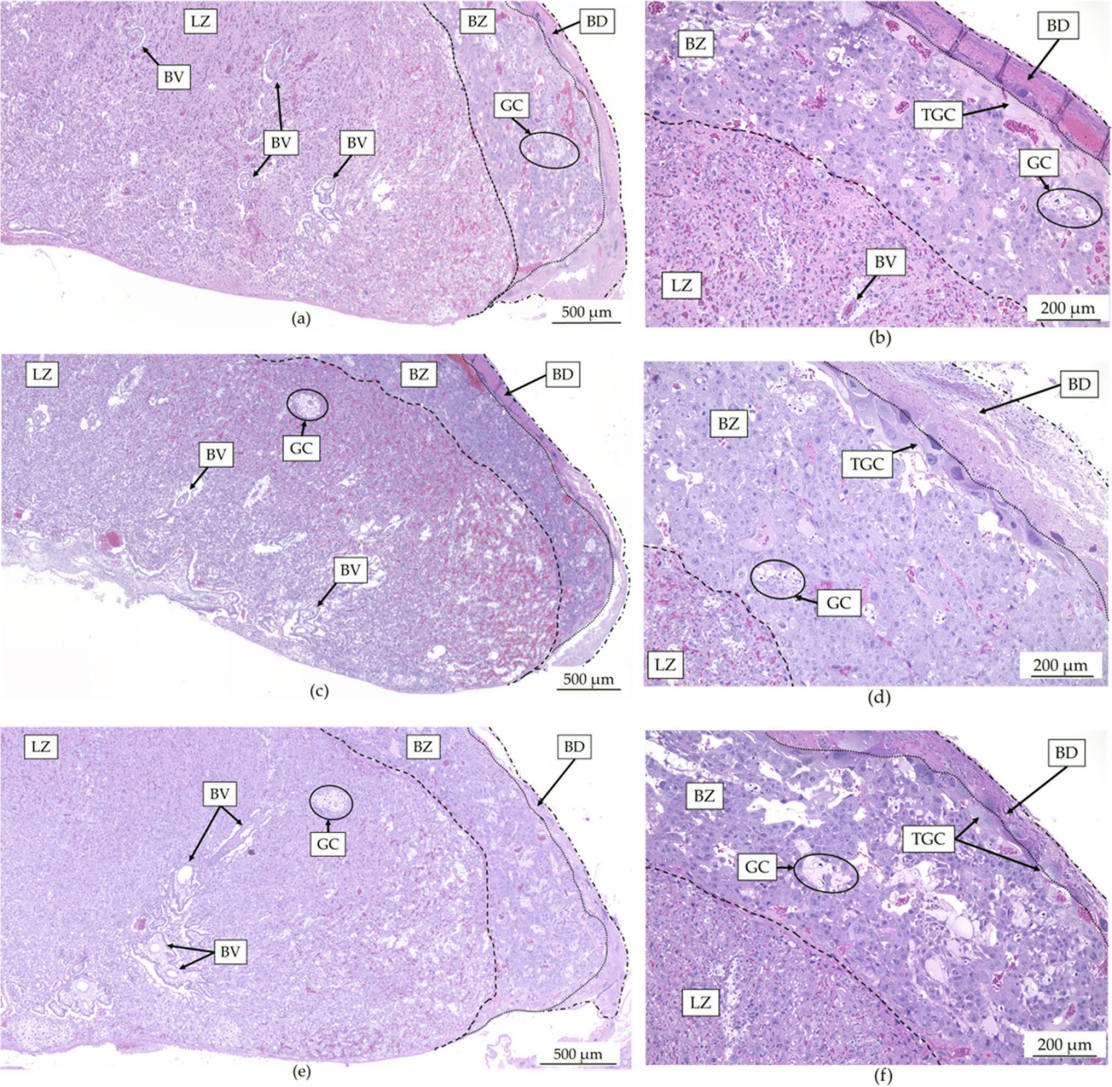
Histologic micrographs of rat placenta on gestational day 19. The dotted line delimits a placental zone. **a** Representative staining of placental slices at 4X from the control group; **b** representative staining of placental slices at 10× from the control group; **c** representative staining of placental slices at 4× from the 2.5 mg fluoride/kg/d; **d** representative placenta slice staining at 10× of the 2.5 mg fluoride/kg/d group; **e** representative placenta slice staining at 4× of the 5 mg fluoride/kg/d group; **f** representative placenta slice staining at 10× of the 5 mg fluoride/kg/d group; H&E stain, BV, blood vessel; BD, basal decidua; GC, glycogenic cells; TGC, trophoblastic giant cells; BZ, basal zone; LZ, labyrinth zone

In addition, Fig. 5 shows representative micrographs of the three experimental groups, a panoramic image of the tested blood vessels and a close-up of the same sections to differentiate the placental zones (delimited by dotted lines) and some cell lines characteristic of the placental zone e.g. the LZ contains syncytiotrophoblast and cytotrophoblast, and the BZ is characterised by the presence of clusters of GC and trophoblast giant cells (TGC).

### Effects on the Placental Vasculature

**Fig. 6:**
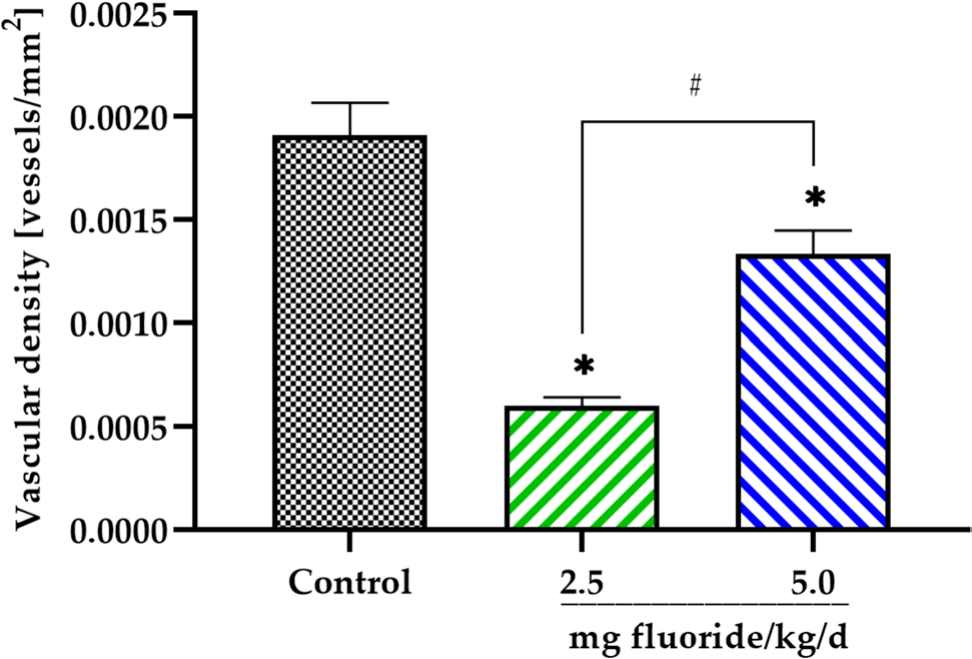
Impact of maternal fluoride exposure on vascular density in the labyrinthine zone area. Bars represent mean ± SD of vascular density expressed in vessels/mm^**2**^. *p < 0.0001 significantly different from the control group (one-way ANOVA test with post hoc Tukey test). #*p* < 0.0001 significantly different between fluoride exposure groups (one-way ANOVA test)

To investigate whether vasculature of placenta was affected by fluoride exposure, the vascular density of the labyrinth area (VDZLa) was assessed. Our results show a marked decrease (*p* < 0.0001) in vascular density in the fluoride treated groups compared to the control. A significant difference was also observed between the 2.5 and 5 mg fluoride/kg/day groups (*p* < 0.0001) (Fig. 6).

**Fig. 7:**
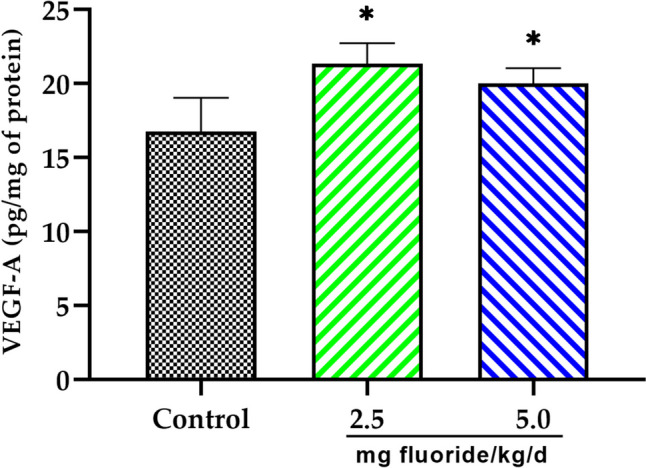
Vascular endothelial growth factor (VEGF-A) concentration in placental tissue. Bars represent mean ± SD of VEGF-A concentration expressed in pg/mg of protein. *p < 0.05 significantly different from the control group (one-way ANOVA test with post hoc Tukey test)

Finally, we examined the VEGF-A concentration in the placental tissue of the fluoride exposure groups. VEGF-A is an important factor involved in signalling pathways for vascular maintenance in placenta. Our finding revealed a statistically significant increase in VEGF-A concentration among the groups exposed to fluoride compared to the control group (Fig. 7). This increase was greater in the 2.5 mg fluoride/kg/d group (*p* < 0.05) than in the 5 mg fluoride/kg/d group (*p* < 0.05).

## Discussion

The intrauterine effects of fluoride exposure are not well understood. In our study, we evaluated the potential placental alterations of fluoride exposure on a rat model that simulated exposure conditions prior to conception and during pregnancy in endemic areas. Our main finding is that fluoride exposure produced significant alterations in regard to foetal-placental weight ratio and placental morphology, as well as variations in vascular density and placental concentrations of VEGF-A. These effects exhibited distinctive differences depending on the dose of fluoride exposure, suggesting the possible activation of a different associated mechanism.

This study evaluated placental alterations on a rat model of biologically relevant oral fluoride concentrations. The concentrations of fluoride used were lower than the reported no-observed-adverse-effect level (NOAEL) for reproductive and developmental effects in Wistar rats, which is 8.5 to 13.7 mg fluoride/kg/d [19]. The concentrations used in the present study consist of a fluoride exposure of 21 and 42 ppm in water. In rodents, fluoride is distributed and cleared 5–10 times faster than in humans, and the effects are less toxic [20, 21]. Thus, the concentrations used in this study would be equal to human exposures at concentrations of 2.1 and 4.2 ppm, which can be found naturally in water contaminated with fluoride [22].

Placental efficiency, quantified by the ratio of foetal or birth weight to placental weight, is often used as a measure of placental efficiency in humans and animals and can be used as a proxy for how the placenta has adapted to meet foetal nutritional requirements [23, 24]. It is well recognized that both low or high changes in placental efficiency were associated with negative foetal development outcomes such as intrauterine growth restriction (IUGR), foetal death and low Apgar scores [23, 25]. In our study, opposite results regarding the weight and height of foetuses were noticed between the two doses of fluoride exposure compared with the control group. The decreased ratio of foetal/placental weight and the noncorrelation of them in the group exposed to 2.5 mg fluoride/kg/d could indicate a failure of placental efficiency, which implies that there may be a decrease in placental nutrient transfer. On the other hand, while this relationship was reestablished within the 5 mg fluoride/kg/d group, the increased foetal/placental weight ratio and the presence of erythema in the exposed foetuses lead us to hypothesise that the apparent increase in “efficiency” reflects a possible occurrence of inflammatory process in the foetus, rather than a genuine biological efficiency [23]. On the other hand, previous experimental studies that investigated the effects of intrauterine fluoride exposure, yielded inconsistent results on foetal outcomes such as foetal weight and height [6, 26]. These variations in outcomes, when compared to our findings, could potentially be attributed to differences in the dose of fluoride exposure employed in these studies. Furthermore, it is worth noting that none of these previous studies have specifically investigated alterations of fluoride at the placental level directly. Nonetheless, it is evident that further research is needed to provide a more compressive effect of fluoride on placental function.

To better understand the placental changes associated with fluoride exposure, we conducted an analysis focusing on some structural characteristics. In our study, we observed an increase in placental weight and changes in the thickness of the placental areas. The placental thickness is a nonspecific finding, however, variations in both reductions and increases have been associated with negative pregnancy outcomes caused by exposure to several toxicants that disrupt placental structure on multiple levels [27–31]. Our findings showed that exposure to 5 mg fluoride/kg/d increases the thickness of BD and LZ, while the exposure to 2.5 mg fluoride/kg/d only increases the thickness of BZ. Experimental studies have shown that under conditions of sustained damage to the placenta, the BD zone spongiotrophoblast proliferates and migrates towards both the BZ and the LZ, resulting in the thickening of the placental zones [32]. This migration serves as a compensatory mechanism to offset the reduction in nutrient exchange and maternal blood flow to attend foetal demands and potentially mitigate the effects of impaired foetal growth [27, 32, 33]. It is noteworthy that the observed increase of thickness in placental zones is mainly attributed to hormonal imbalances, given that these zones are regulated by hormones [34]. Furthermore, it seems probably that the ultrastructural changes in our study are connected to the distinct relationship between foetal and placental weights observed in response to each dosage of fluoride exposure. This, in turn, may be attributed to the activation of varying mechanisms of fluoride toxicity and placental adaptation processes. Nonetheless, further research is necessary to elucidate this relationship. In addition, the fluoride capacity to induce placental adaptation mechanisms could be explained through the generation of oxidative stress or through the reduction of oestrogen levels [35, 36]. These mechanisms have the potential to generate structural and morphological changes in the placenta, which may serve as part of a compensatory mechanism [37].

Optimal placental vascularization has been related to the establishment of a successful pregnancy and its alteration has been directly associated with changes in placental function and the activation of adaptive mechanisms. Our findings showed a decrease in the VDLZa and an increase in placental concentration of VEGF-A in the fluoride exposure groups, both parameters associated with placental vasculature. It is important to note that regions exhibiting decreased vascular density correspond to regions characterised by compromised maternal perfusion and inadequate oxygenation in utero [38]. Importantly, these variations in vascular density can be associated with alterations in molecules that mediate placental vascular maintenance, including VEGF-A. This factor plays an important role in vasculogenesis and angiogenesis, and its imbalance has been linked with hypoxia and adverse outcomes such as preeclampsia [39, 40]. In this context, our results suggest that fluoride exposure could affect placental vasculature, potentially resulting in an impact on placental efficiency and function.

Oxidative stress is continuously generated in the placenta, but its overproduction can damage biomolecules, accelerating placental insufficiency and therefore compromising foetal viability [15]. MDA is a product of lipid peroxidation and has been widely used as an oxidative damage marker. The increase of MDA levels associated with oxidative stress generation after fluoride exposure has been reported in several studies [8]. However, in our study, we only observed a significant increase in placenta concentration of MDA in the group exposed to 2.5 mg fluoride/kg/d. We hypothesised that different results in the 5 mg fluoride/kg/d group might be caused by the activation of different toxicity mechanisms or the activation of adaptive mechanisms. The decrease of oxidative damage characterised by a decrease in the levels of MDA in placenta tissue has previously been reported in obese and diabetic pregnancies; these studies have suggested the activation of protective or adaptive mechanisms that prevent oxidative damage produced, such as nitrosative stress [41, 42]. On the other hand, Chouhan and Flora [43] have reported that oxidative stress mediates fluoride toxicity at low levels of fluoride exposure in soft tissues but not in higher doses. This suggested that different modes of action depend on the fluoride exposure dose. This could also explain the absence of a dose–response relationship observed in placental MDA concentration with increased fluoride exposure.

In summary, our findings showed a differential response of the evaluated placenta parameters to fluoride exposure. This suggests that fluoride may affect foetal development through a biphasic dose–response, where adaptive mechanisms and different mechanisms of fluoride-induced toxicity, are both activated. A research conducted by Ortiz-García and colleagues has reported a nonlinear association of maternal urinary fluoride concentration with birth weight [44]. The study demonstrated that the association of fluoride exposure with changes in birth weight was dependent on the trimester of pregnancy and the breakpoints of the fluoride exposure levels. These findings also suggest a possible dual effect of exposure to different levels of fluoride during pregnancy. The biphasic effects of fluoride have been recognized in other organs and could be linked with the activation of different mechanisms of toxicity [10, 43, 45]. However, as we discussed previously, we do not discard the potential activation of adaptive mechanisms in response to fluoride-induced damage [24].

Alternatively, it has been reported that fluoride can induce systemic vascular alterations such as atherosclerosis and blood pressure modification [46, 47]. It is plausible that those systemic effects may also be present during pregnancy and potentially contribute to alterations in the placenta and umbilical cord, leading to modifications of foetal blood flow [48, 49]; however, this has not yet been studied. Recently, the effect of fluoride exposure on human intrauterine development has gained importance. Clearly, our understanding of the mechanisms associated with placental alterations of fluoride exposure, as well as the potential effects on development and growth outcomes after birth remains incomplete and requires further investigation. Nevertheless, this study reinforces the findings that exposure to fluoride during pregnancy could affect foetuses [6, 44] and offers important insights that can be helpful to guide further research.

To our knowledge, this is the first study to propose a model of fluoride exposure conditions during pregnancy to identify placental and foetal alterations. Nevertheless, we acknowledge that our study has limitations. First, at the vascular level, we limited ourselves to counting the number and determining the VDLZa; however, we did not evaluate the vascular damage. Second, at a methodological level, we limited ourselves to determining placental weight and length, while there are other parameters, such as Doppler velocimetry, of the impedance of blood flow in the umbilical artery that we could have used to determine whether these changes in placental and foetal weights were due to poor placental perfusion [50, 51]. Last, placenta structures in rats may be more resistant to placental disruption than placental structures in humans due to rats’ resistance to fluoride exposure [52]. Thus, whether fluoride can cause alterations in placentation, defects in the contractility of the placental vasculature, apoptosis of invading cytotrophoblasts, or inadequate remodelling of spiral arteries, alterations that are well known to lead to decreased placental blood flow [53], which is associated with the development of pathophysiological processes, remains to be studied.

## Conclusions

In conclusion, the results of this study showed that exposure to environmentally relevant concentrations of fluoride produces significant non-monotonic changes in the foetal-placental weight ratio, affects the thickness of the placental area, increases VEGF-A levels, and causes a decrease in the VDLZa. In addition, we observed an inadequate foetal growth in both doses and only an increase of MDA in the placenta at lower exposure dose. These findings are indicative of alterations in both placental morphology and efficiency, modifications that are related to later developmental defects. This study deepens the knowledge of the effects of fluoride toxicity on the placenta and its impact on foetuses and provides new avenues for the study of the effects of toxicity during gestation.
